# Crosstalk Between Cancer Associated Fibroblasts and Cancer Cells in the Tumor Microenvironment After Radiotherapy

**DOI:** 10.1016/j.ebiom.2017.03.004

**Published:** 2017-03-04

**Authors:** Genichiro Ishii

**Affiliations:** Division of Pathology, Exploratory Oncology Research & Clinical Trial Center (EPOC), National Cancer Center, 6-5-1, Kashiwanoha, Kashiwa-City, Chiba 277-8577, Japan

Cancer tissue is composed of not only cancer cells, but also non-cancerous cells known as stromal cells, which create a specific tumor microenvironment. The biological importance of the tumor microenvironment was first proposed by Paget in 1886 and his “seed and soil” theory (Paget, 1886) has become widely accepted. However, many studies have focused on only the cancer cells (“seed”) and its key molecular mechanism and functional signaling pathway in tumor development and progression, which have been extensively characterized. In contrast, the importance of microenvironment (“soil”) is poorly understood, possibly because of its structural and functional complexity.

The most abundant cell types among stromal cells are fibroblasts (also known as cancer-associated fibroblasts; CAFs), which reportedly play important roles in several aspects of the chemotherapeutic process (Wang et al., 2009, Yoshida et al., 2015). The molecular interaction between cancer cells and CAFs may be a key in the regulation of resistance to cancer cell-targeted chemotherapy. Therefore, many possible therapeutic strategies targeting this crosstalk have been proposed. Radiotherapy is one treatment option along with chemotherapy; however, microenvironmental factors that affect the resistance of cancer cells after irradiation are not fully understood. Certain subpopulations of cancer cells, such as cancer stem cells, are intrinsically radioresistant (Pajonk et al., 2010), while it is speculated that CAFs also transmit extrinsic signals to survive and to regrow irradiated cancer cells. It will be challenging to identify the specific interactions between cancer cells and CAFs in the tumor microenvironment after radiation therapy.

In this issue of *EBioMedicine*, Wang et al. (2017-in this issue) clarified the novel roles of CAFs in post-radiotherapy recurrence. Using a mouse model, they first found that CAFs promoted the regrowth of irradiated cancer cells by secreting IGF1, IGF2, and CXCL12. They further revealed that these secreted factors derived from CAFs increased reactive oxygen species (ROS) levels in irradiated cancer cells, enhancing PP2A activity and increasing autophagy accompanied by the repression of mTOR activation. Moreover, using a neutralizing antibody against IGF2 and an autophagy inhibitor, Wang and his colleagues demonstrated the possibility of targeting the autophagy pathway as a promising strategy for making cancer cells more sensitive to radiation. This is particularly important from a clinical perspective because radiotherapy cannot kill all cancer cells and surviving radioresistant cells can re-proliferate to form a large cancer mass. Understanding how microenvironmental factors promote radioresistance would develop new treatment paradigms in cancer control after radiotherapy (Barker et al., 2015).

Autophagy enables cancer cell survival by maintaining energy production, which can lead to therapeutic resistance (Yang et al., 2011). Several preclinical studies showed that clinically relevant doses of radiation directly promote autophagy in cancer cells and inhibition of autophagy can restore chemosensitivity and enhance cancer cell death (Gewirtz, 2014). Although the current evidence presented by Wang and colleagues also shed light on the importance of autophagy in the field of radiotherapy, the mechanism proposed by them was more complicated. The authors showed that via soluble factors, irradiated CAFs activate autophagy in irradiated cancer cells, indicating the importance of the crosstalk between CAFs and cancer cells even in the tumor microenvironment after radiotherapy. This finding strongly suggests that targeting autophagy has potential benefits for radiotherapy patients.

Stereotactic body radiation therapy (SBRT) is a treatment modality that enables delivery of higher doses to the cancer mass without increasing doses to the surrounding tissue. In many human cancer tissues, CAFs were observed not only within the cancer mass, but also at the peritumoral area, distal to the outer margin of the cancer. Therefore, there is a high probability that SBRT cannot damage CAFs in the peritumoral area. The authors indicated that the frequency of recurrence in patients with SBRT treatment was higher than that in patients with external beam radiotherapy. Taken together, SBRT may be beneficial for reducing normal tissue damage; however, these clinical and experimental results suggest that this therapy should be used with caution.

It is well-known that irradiation can induce biological changes in CAFs (Hellevik et al., 2012). CAFs have heterogeneous origins, phenotypes, and functions within the tumor microenvironment; therefore, CAFs should be classified into subpopulations for functional analysis (Ishii et al., 2016). Thus, the authors' work raises some further questions such as whether all subpopulations of CAFs induce autophagy in irradiated cancer cells. Further studies should examine which subpopulation of CAFs promotes autophagic activity in irradiated cancer cells.

Tumor relapse after radiotherapy is a serious problem. Here, Wang and colleagues have clearly demonstrated that CAFs do not act as “enemies” but as “friends” of irradiated cancer cells, by modulating autophagic activity. The present study provides interesting perspectives for understanding the tumor microenvironment after radiotherapy and provides new treatment opportunities for cancer patients undergoing radiation therapy.

## Conflict of Interest

The author declares no conflicts of interest.
